# Supplementary material for: Modulation of Reoviral Cytolysis (I): Combination Therapeutics
Source: Viruses. 2023 Jun 29;15(7):1472. doi: 10.3390/v15071472 (PMC10385176; doi:10.3390/v15071472)
Supplement: Supplementary file 1 [file viruses-15-01472-s001.zip › viruses-2461611-supplementary.pdf]

Supplementary Figure S1 a,b

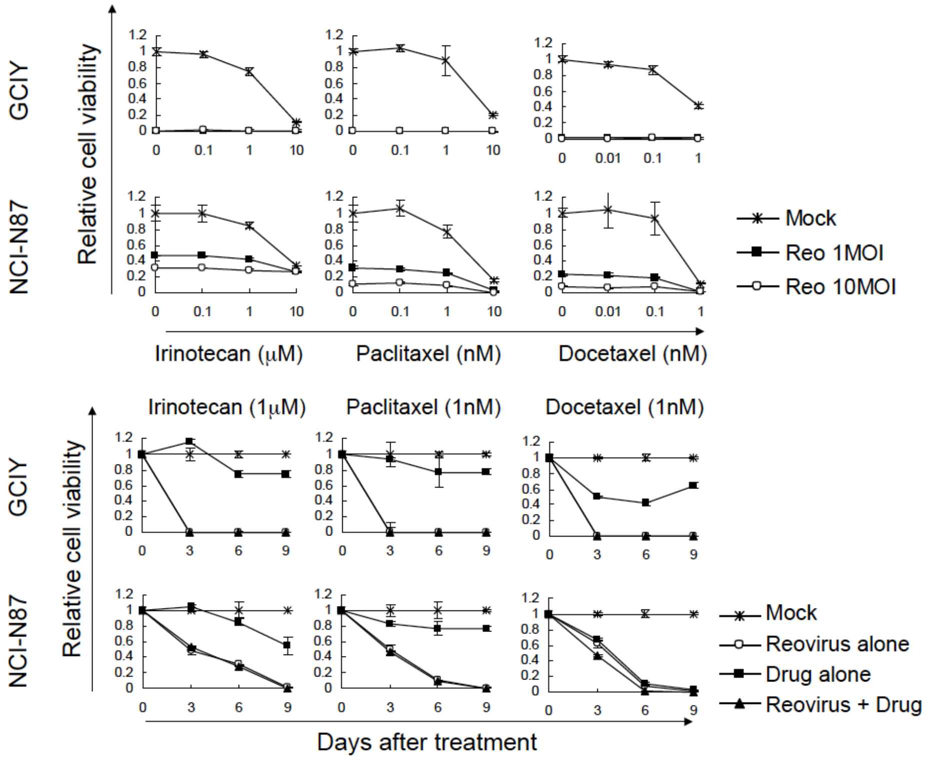

Combination effects of reovirus and chemotherapeutic agents (irinotecan, paclitaxel, docetaxel) on human gastric cancer cell lines (GCIY, NCI-N87). *a*) Cells were infected with 1 or 10 MOI of reovirus and exposed to chemotherapeutic agents at the indicated concentrations. Cell viability was assessed by WST-1 assay at 6 days after treatment. Bars, standard deviation (SD). *b*) Time course of the combined effect of reovirus plus chemotherapeutic agents on gastric cancer cell lines. Cells were treated with 10 MOI of reovirus, chemotherapeutic agent (1μM irinotecan, 1nM paclitaxel, 1nM docetaxel), or a combination of both, and cell killing efficacy was evaluated by WST-1 assay over 9 days.

Supplementary Figure S2 a,b

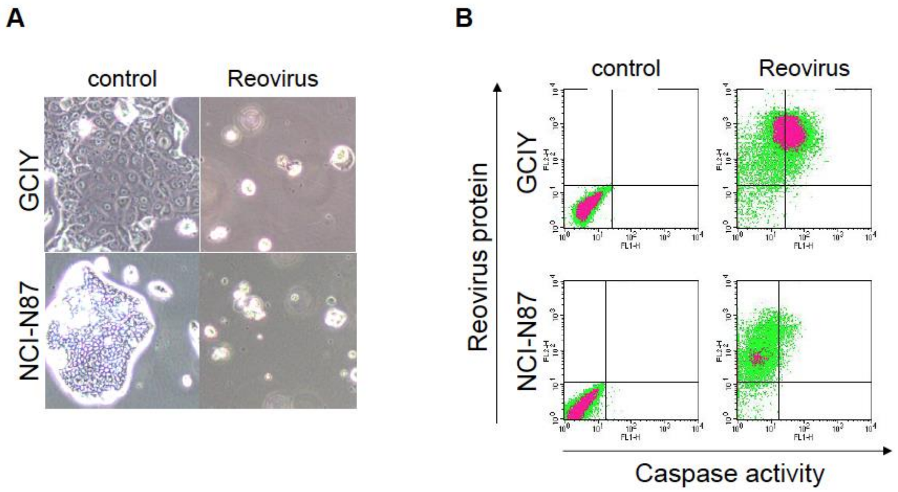

a) Cytopathic effects of reovirus in GCIY and NCI-N87. Cells were infected with 10 MOI of reovirus and photographed 5 days after treatment. ×100 magnification. b) Caspase

activation and reovirus protein expression were determined by flow cytometry in gastric cancer cell lines, GCIY and NCI-N87. Cells were treated with 10 MOI of reovirus, and caspase activation and reovirus protein expression were evaluated by flow cytometry at 5 days post treatment.

Supplementary Figure S3

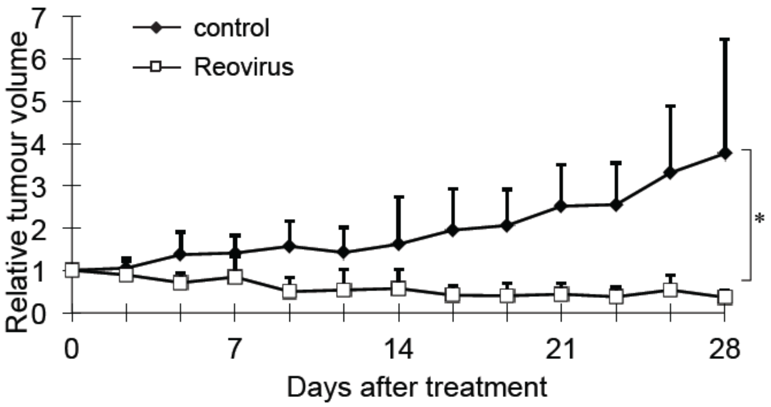

Antitumor effects of intratumourally injected reovirus against established flank GCIY xenograft tumours in nu/nu mice. GCIY cells (2×10^{6} cells/each) were subcutaneously injected into the left flanks of mice. Reovirus was administered at 1×10^{8} pfu/body. Five mice were used for each group. Tumors were measured every 2 or 3 days, and tumour volume was expressed relative to volume at commencement of treatment. Statistical significance was defined as $p<0.05$ (*) (Mann-Whitney *U* test). GCIY gastric cancer cells were very effectively killed by reovirus alone *in vitro* (Fig. 1a) and *in vivo*.
